# Supplementary material for: Childhood socioeconomic position relates to adult decision-making: Evidence from a large cross-cultural investigation
Source: PLoS One. 2024 Nov 12;19(11):e0310972. doi: 10.1371/journal.pone.0310972 (PMC11556711; doi:10.1371/journal.pone.0310972)
Supplement: S1 Text — (DOCX) [file pone.0310972.s001.docx]

# **Supplementary materials**

As outlined in the introduction, the experience of lower socioeconomic position can vary substantially depending on the country and region you live in. Ruggeri et al. [[S1]](https://www.zotero.org/google-docs/?ynR3Qz) provided an extensive between country analysis of the variation in temporal discounting as a result of contemporaneous country-level factors such as inequality and gross domestic product in the same sample of participants. However, given our focus here on developmental factors and the potential for variation in our results by country, we conducted a series of additional analyses to fully utilize this multinational dataset and probe the robustness of our results, specifically at the country level. The first section includes a series of figures from our initial exploration of the dataset followed by a brief interpretation and explanation of the rationale motivating our analysis. We then describe three sets of analyses. Specifically, we include 1.) graphs plotting country-level differences in the relationship between childhood socioeconomic position and temporal discounting; 2.) examination of the robustness of main effects between Western, educated, industrialized, rich, democratic nations (W.E.I.R.D) and non-W.E.I.R.D countries; 3) between**-**country analysis of main effects with the global extreme poverty in participants’ year and country of birth included as a covariate.

## Exploratory analyses

**S1 Figure:** Distributions of temporal discounting broken down by country.

**S2 Figure.** Within country relations between childhood socioeconomic position and temporal discounting.

## Supplemental analyses

### Between country results

To examine the relationship between temporal discounting and childhood socioeconomic position, we constructed a multiple regression model with TD score as the dependent variable and country, employment status, age, childhood socioeconomic position, and recent economic change all included as covariates. This was done for each country separately, meaning 61 statistical models were constructed. In these analyses, we find that participants in ~95% of the countries surveyed showed the relation noted in our main manuscript (i.e., lower childhood socioeconomic position was related to greater discounting). Relations were not seen in Ghana, Turkey, and Ethiopia and only at “*trend*” levels in those three countries (as shown in Figure S3).

**S3 Figure.** Forest plot showing the coefficient estimate for childhood socioeconomic position in every country represented by the sample.

### Western, educated, industrialized, rich, democratic nations

In recent years, concerns about Western, educated, industrialized, rich, democratic (WEIRD) samples and generalizability in the behavioral sciences have mounted. The WEIRD framework characterizes countries that share similar values and societal structures. Participants from WEIRD countries have been overrepresented in academic studies. Findings that were once considered “universal” have failed to replicate in multicultural, non-WEIRD samples. To investigate the generalizability of our results, we completed sensitivity analyses examining the two and three-way interactions between WEIRD status, childhood SEP, and recent economic change.

To categorize WEIRD and non-WEIRD countries, we followed a procedure implemented by Klein et al. that assigns a “WEIRDness” score to each country of origin [[S2]](https://www.zotero.org/google-docs/?cUbaHk). Western countries were given a score of 1 and Eastern countries a score of 0 based on the Brandt Line [[S3]](https://www.zotero.org/google-docs/?f3os2B). Each country was assigned an Education score based on their education index sub-score from the United Nations Human Development Index [[S4]](https://www.zotero.org/google-docs/?df1g0P). Countries with industrialized economies were scored as a 1 and developing and emerging economies as 0 based on the United Nations Industrial Development Report 2022 (Annex C) [[S5]](https://www.zotero.org/google-docs/?S0hhBr). Rich countries were those labeled as “developed” in the United Nations World Economic Situations and Prospects report and were assigned a 1 or 0 [[S6]](https://www.zotero.org/google-docs/?relbsK). A democracy index calculated by the Economic Intelligence Unit was used to assign a score for Democracy to each country [[S7]](https://www.zotero.org/google-docs/?r16M4a). Then, the mean of the five variables was calculated to produce a WEIRDness score for each country. Finally, a classification of WEIRD (1) or non-WEIRD (0) was made based on whether the WEIRDness score fell above or below the median WEIRDness score.

Similar to the linear mixed-effects models in the main manuscript, our model included temporal discounting as the dependent variable, and gender, age, employment status, highest level of education completed, and two- and three-way interactions between childhood socioeconomic position, recent economic change, and a WEIRD binary indicator. Interestingly, and as shown below (in Table S1), we see the same main effects and interactions as the main manuscript (Significant Main Effects: Recent Economic Change and Childhood Socioeconomic Position; Significant Interaction: Recent Economic Change x Childhood Socioeconomic Position). However, we also see a main effect of WEIRD status (p<0.001), but no significant interactions with WEIRD status, recent economic change, and childhood socioeconomic position (all p’s > 0.318).

**Table S1.** **Main and interaction effects of WEIRD and childhood socioeconomic position on temporal discounting.**

|  | **Temporal discounting** | | | |
| --- | --- | --- | --- | --- |
| *Predictors* | *Estimates* | *CI* | *p* | |
| (Intercept) | 0.52 | 0.39 – 0.65 | | **<0.001** |
| Employment (Unemployed) | 0.01 | **-**0.03 – 0.04 | | 0.732 |
| Age | 0.03 | 0.01 - 0.04 | | **0.001** |
| Gender (Male) | 0.03 | **-**0.00 - 0.06 | | 0.091 |
| Recent Economic Change | **-**0.06 | **-**0.09 – **-**0.04 | | **<0.001** |
| WEIRD | **-**0.64 | **-**0.82 – **-**0.46 | | **<0.001** |
| Childhood Socioeconomic Position | **-**0.08 | **-**0.14 – **-**0.02 |  | **0.012** |
| WEIRD x Childhood Socioeconomic Position | 0.02 | **-**0.07 – 0.11 | | 0.601 |
| Recent Economic Change x WEIRD | **-**0.01 | **-**0.04 – 0.02 | | 0.564 |
| Recent Economic Change x Childhood Socioeconomic Position | 0.03 | 0.00 – 0.05 | | **0.016** |
| Recent Economic Change × WEIRD × Childhood Socioeconomic Position | **-**0.01 | **-**0.04 – 0.01 | | 0.318 |
| **Random Effects** | | | | |
| s^2^ | 0.76 | | | |
| t_00_ | 0.10_Country_ | | | |
| ICC | 0.11 | | | |
| N | 61_Country_ | | | |
| Observations | 12950 | | | |
| Marginal R^2^ / Conditional R^2^ | 0.129 / 0.227 | | | |

### Global extreme poverty rate

Obviously, there are significant variations in socioeconomic conditions between countries. Low socioeconomic position can mean very different things depending on the country. To understand this, we examined if the global extreme poverty rate for each participant's birth country at the year of their birth was related to adult temporal discounting. For these analyses, we gathered economic disparity data near the year of each participant’s birth in their country of residence from the Clio Infra project ([https://clio-infra.eu/index.html#](https://clio-infra.eu/index.html)). We chose to operationalize rates of extreme poverty with data on Global Extreme Poverty as defined by the dollar a day (DAD) estimate (or Global Extreme Poverty DAD). This approach involves using a single global poverty line based on the distribution of household income or consumption expenditure [[S8]](https://www.zotero.org/google-docs/?CtntyJ). The extreme poverty rate represents the share of the people in a country below the poverty line. It is expressed as a share from 0-1. With this metric, data was missing for 2.7% of our sample, so we imputed values using random forests, multiple imputation by chained equations via the mice package in R. Specifically, missing values were imputed based on all of the observed data, as well as six historical measures of global inequality (child mortality by country income level, GDP per capita, gini coefficient, gini wealth coefficient, gross domestic product, and life-expectancy at birth, all pulled from <https://ourworldindata.org/>). For each measure, the value in the participant’s birth year and country was used. We constructed 100 imputed data sets and pooled statistical models accordingly. To probe the robustness of our results, we reconstructed our main effects model with the inclusion of Global Extreme Poverty DAD as a covariate. Below in Table S2 are the results of the model.

**Table S2.** **Main effects of childhood socioeconomic position on temporal discounting with the Global Extreme Poverty Rate added as a covariate**

|  | **Temporal discounting** | | | |
| --- | --- | --- | --- | --- |
| *Predictors* | *Estimates* | *CI* | *p* | |
| (Intercept) | **-**0.02 | **-**0.13 - 0.10 | | 0.789 |
| Employment (Self-employed) | 0.11 | 0.06 - 0.16 | | **<0.001** |
| Employment  (Part-time) | 0.04 | -0.02 - 0.09 | | 0.183 |
| Employment  (Looking) | 0.20 | 0.13 - 0.28 | | **<0.001** |
| Employment  (Personal reasons) | 0.08 | 0 - 0.15 | | **0.045** |
| Employment (Full-time student) | **-**0.01 | **-**0.06 - 0.04 | | 0.630 |
| Employment (Retired) | 0.07 | **-**0.03 - 0.18 | | 0.166 |
| Age | 0.02 | 0.00 - 0.04 | | **0.033** |
| Gender (Male) | 0.02 | **-**0.01 - 0.05 | | 0.244 |
| Gender (Other) | **-**0.03 | **-**0.13 - 0.06 | | 0.456 |
| Global Extreme Poverty Rate | **-**0.02 | **-**0.05 - 0.01 | | 0.251 |
| Childhood Socioeconomic Position | **-**0.02 | **-**0.04- -0.01 | | **0.006** |
| **Random Effects** | |  | |  |
| s^2^ | 0.76 |  | |  |
| t_00_ | 0.21_Country_ |  | |  |
| ICC | 0.21 | | | |
| N | 61_Country_ | | | |
| Observations | 13323 | | | |
| Marginal R^2^ / Conditional R^2^ | 0.005 / 02.16 | | | |

Of note, and contrary to our predictions, country-level indicators of historical hardship and inequality did not directly relate to adult discounting. We will be cautious in interpreting this null effect, as these historical indicators had a fair deal of missingness necessitating data imputation. While greater discounting has been found in countries with greater current economic inequality [[S1]](https://www.zotero.org/google-docs/?dX5mqs), future work, especially prospective longitudinal studies, could be powerful in understanding if societal, as opposed to individual, inequality may shape decision-making. However, if similar associations between societal-level conditions in childhood and adult decision-making are not found by other research groups, this would raise a number of interesting questions related to objective versus subjective experiences and what might be more robustly influencing behavior. Emerging work suggests that self-perceptions of experiences (rather than objective measures) may be more powerful predictors of critical life outcomes. For example, youth who perceived their neighborhood to be unsafe were two times more likely to report serious psychological distress, compared to those who perceived greater neighborhood safety [[S9,S10]](https://www.zotero.org/google-docs/?Ydkb03). This has also been found for stress exposure and parenting [[S11]](https://www.zotero.org/google-docs/?44u6rs) . Examined collectively, it may be that perceived inequities in childhood may play an important role in shaping psychological processes associated with temporal decision-making.

### Categorical treatment of childhood socioeconomic position

Finally, we report sensitivity analyses showing the main effect of childhood socioeconomic position on TD when measured categorically rather than continuously. For these analyses, we constructed two sets of models. Similar to the main manuscript, we first made linear mixed effect models with temporal discounting as the dependent variable and employment, age, gender, and childhood SEP (as a categorical variable, with Poor group as the reference); country was included as a random factor. In these models, the “Poor” group showed greater discounting “Below average but not poor” group (β=-0.07790, t=-2.41334, p=0.015, d=-0.0425), the “Around average” group (β=-0.06482, t=-2.111, p=0.0347, d=-0.037), “Above average but not wealthy” group (β=-0.10391, t=-3.286, p=0.00102, d=-0.0578), and “Wealthy” group (β=-0.10464, t=-2.245, p=0.024, d=-0.0395). These results are shown in Figure S4. In a second set of models, we also included education as an additional independent (control) variable, still including employment, age, gender, and childhood SEP (as a categorical variable, with Poor group as the reference). In these models, the “Poor” group showed greater discounting “Below average but not poor” group (β=-0.0675, t=-2.091, p=0.036, d=-0.0403) and “Above average but not wealthy” group (β=-0.0855, t=-2.696, p=0.00102, d=-0.0475). There was however no difference between the “Poor” group and the “Around average” group (β=-0.0507, t=-1.649, p=0.099) or the “Wealthy” group (β=-0.0824, t=-1.763, p=0.077, d=-0.0311). These results are also shown in Figure S4.

Briefly reflecting on this finding in relation to what was reported in the main manuscript, we see that the lowest childhood SEP group shows greater temporal discounting than the multiple other groups (below average, above average). However, depending on the model covariates, there were variations in differences between the poor and other groups. This suggests that a continuous measure of social position may obscure non-linear relationships that become evident when analyzing this variable categorically. The variability in findings for the “Around average” v. “Poor” groups are surprising, and we do not want to overinterpret this null effect. For the highest (“Wealthy”) group, it is interesting to speculate that greater discounting may be seen in this group because of societal and structural privileges that may protect them from the negative consequences of greater discounting.

**S4 Figure.**

##

##

## References

S[1. Ruggeri K, Panin A, Vdovic M, Većkalov B, Abdul-Salaam N, Achterberg J, et al. The globalizability of temporal discounting. Nat Hum Behav. 2022;6: 1386–1397.](https://www.zotero.org/google-docs/?nSeNM9)

S[2. Klein RA, Vianello M, Hasselman F, Adams BG, Adams Jr RB, Alper S, et al. Many Labs 2: Investigating variation in replicability across samples and settings. Adv Methods Pract Psychol Sci. 2018;1: 443–490.](https://www.zotero.org/google-docs/?nSeNM9)

S[3. Brandt W, others. North South: a programme for survival; report of the independent commission on international development issues. MIT, Cambridge, MA, US; 1980.](https://www.zotero.org/google-docs/?nSeNM9)

S[4. Programme UND. Human Development Report 2023/2024. 2024th ed. United Nations; 2024. Available: https://www.un-ilibrary.org/content/books/9789213588703](https://www.zotero.org/google-docs/?nSeNM9)

S[5. Organization UNID. Industrial Development Report 2022. 2022nd ed. United Nations; 2022. Available: https://www.un-ilibrary.org/content/books/9789210011501](https://www.zotero.org/google-docs/?nSeNM9)

S[6. Nations U. World Economic Situation and Prospects 2024. United Nations Department of Economic and Social Affairs; 2024.](https://www.zotero.org/google-docs/?nSeNM9)

S[7. Democracy index — ourworldindata.org. Economic Intelligence Unit; 2023. Available: https://ourworldindata.org/grapher/democracy-index-eiu?tab=table](https://www.zotero.org/google-docs/?nSeNM9)

S[8. Ravallion M, Datt G, Van de Walle D. Quantifying absolute poverty in the developing world. Rev Income Wealth. 1991;37: 345–361.](https://www.zotero.org/google-docs/?nSeNM9)

S[9. Valente R, Crescenzi-Lanna L. Feeling unsafe as a source of psychological distress in early adolescence. Soc Sci Med. 2022;293: 114643.](https://www.zotero.org/google-docs/?nSeNM9)

S[10. Meltzer H, Vostanis P, Goodman R, Ford T. Children’s perceptions of neighbourhood trustworthiness and safety and their mental health. J Child Psychol Psychiatry. 2007;48: 1208–1213.](https://www.zotero.org/google-docs/?nSeNM9)

S[11. Kahhalé I, Barry KR, Hanson JL. Positive parenting moderates associations between childhood stress and corticolimbic structure. PNAS Nexus. 2023;2: pgad145.](https://www.zotero.org/google-docs/?nSeNM9)
