# Supplementary figures and images for: Childhood socioeconomic position relates to adult decision-making: Evidence from a large cross-cultural investigation
Source: PLoS One. 2024 Nov 12;19(11):e0310972. doi: 10.1371/journal.pone.0310972 (PMC11556711; doi:10.1371/journal.pone.0310972)

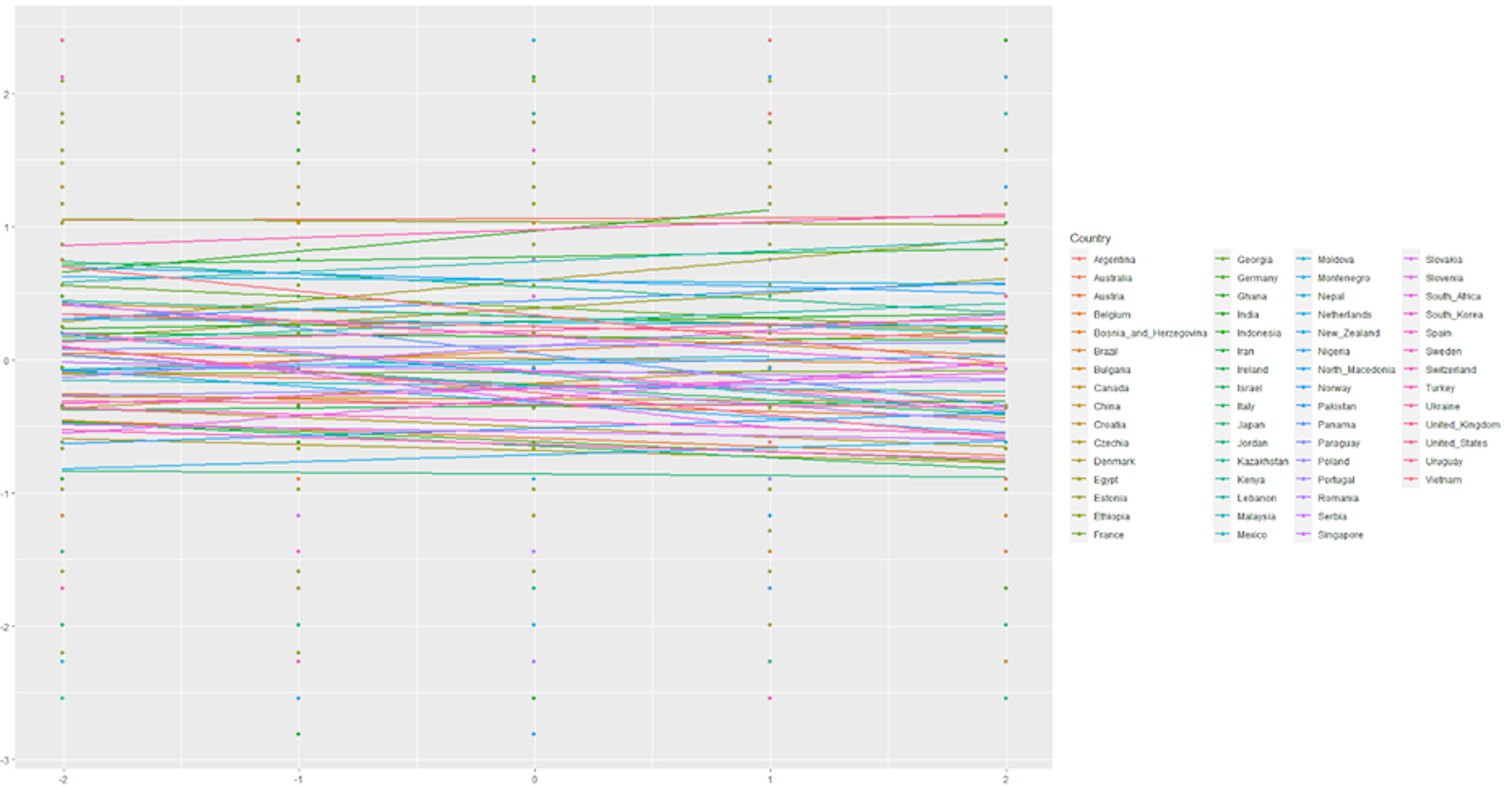

Supplement: S1 Fig — (TIF) [file pone.0310972.s002.tif]

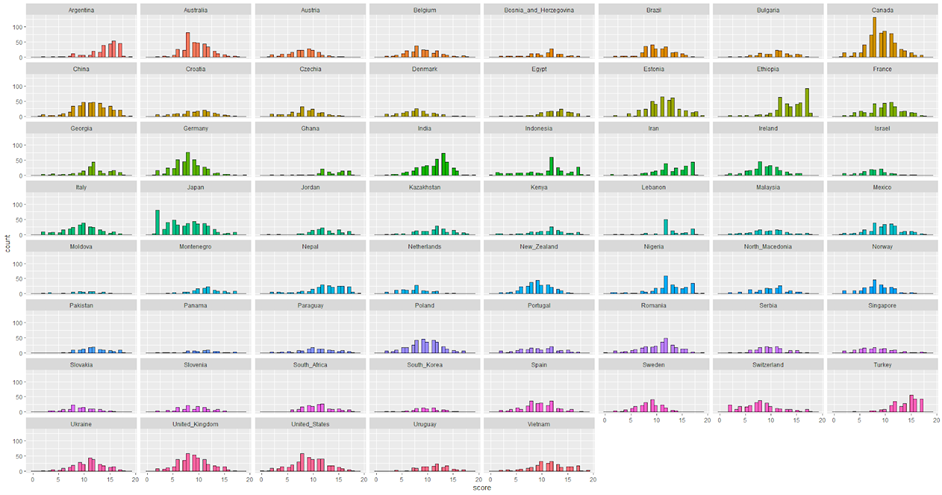

Supplement: S2 Fig — (TIF) [file pone.0310972.s003.tif]

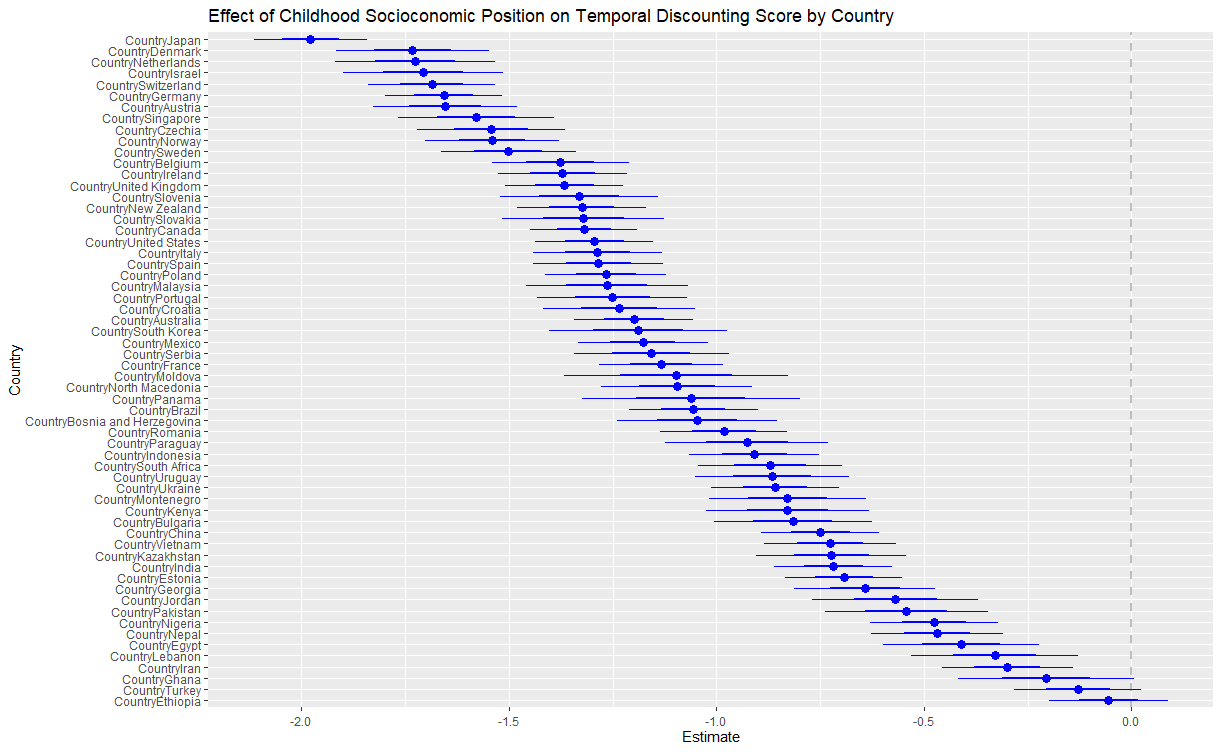

Supplement: S3 Fig — (TIF) [file pone.0310972.s004.tif]

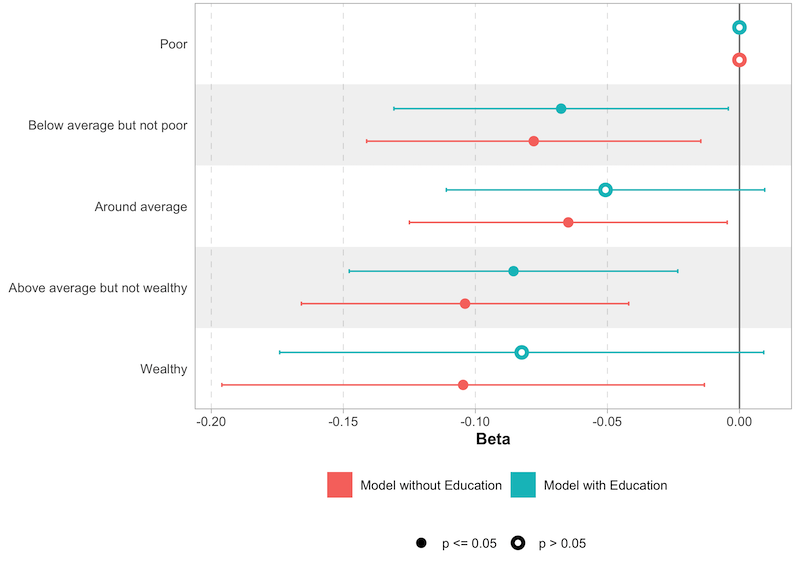

Supplement: S4 Fig — (TIF) [file pone.0310972.s005.tif]
