# Supplementary material for: Childhood socioeconomic position relates to adult decision-making: Evidence from a large cross-cultural investigation
Source: PLoS One. 2024 Nov 12;19(11):e0310972. doi: 10.1371/journal.pone.0310972 (PMC11556711; doi:10.1371/journal.pone.0310972)
Supplement: Attachment — Submitted filename: 01_PLOSOne_Rebuttal_Letter_20240219.docx [file pone.0310972.s006.docx]

Dear Dr. Akaishi,

Thank you very much for the thoughtful critiques that you solicited, and that the reviewers provided for this project. We are very encouraged that the reviewers saw merit in our work. We have revised the manuscript to address all of the points raised in the reviews. To facilitate your evaluation of this revised manuscript, we have reproduced excerpts from each critique in italics, followed by our responses

Reviewer #1

- *The manuscript by Wang and Hanson provides an interesting test of how childhood poverty can influence adult decision-making, particularly temporal discounting*

We thank the reviewer for these positive remarks!

- *My main concern about the paper can also be considered a strength [...] Large datasets are great, and the use of a multinational dataset increases the generalizability of the data. However, what was the effect size? How important is this relationship given the very small beta coefficients? This does not detract from the author’s ideas, but I believe it is critical to understand the importance of these previous experiences to current decision making.*

This is an important point raised by this reviewer. In our revised manuscript, we have tried to provide a much more tempered discussion about the strength of these relations throughout the document. Furthermore, in response to this comment, we have included specific effect size estimates (e.g., Pg. 13), as measured by *Cohen’s d*, for different associations noted in our results section. If there is a preference for a different statistical parameter estimate (Hedges’ g; partial eta), we are happy to include those in future versions of the manuscript.

In addition, given the fact that there are many intervening factors from childhood to adulthood, we believe it is still notable to find relations between childhood SEP and adult temporal discounting. Within our sample, heterogeneous subgroups likely exist, and it would be very interesting to explore if childhood SEP interacted with these intervening factors to predict later adult behavior. We think this is a great direction to pursue in future projects. However, noting these long-term influences on behavior is an important, initial contribution to the literature.

- *In summary, I really liked this paper, but the authors need to indicate the importance of these results. In the beginning of the results, the authors describe the relationships as modest. That is vague. The important point, if possible, is to Even if small, this may be very important and definitely worth reporting. I look forward to reviewing a revised version of this stimulating paper.*

We thank the reviewer for raising this concern. We have added further discussion of this issue in our results section (e.g., Pg. 13).

Reviewer #2

- *The paper is an interesting discussion of an important topic, but as it stands I am not sure I would recommend the current paper for publication for three reasons: First, it does not lay out a clear enough theoretical framework through which to guide the research’s approach, predictions, and findings. Although theoretical contribution is not a key criterion for publication in PLOSOne, it does matter to the extent that the questions and the way of approaching them can be scientifically justified and easily interpreted.*
- *The framing does not enough outline why individual-level decision-making should necessarily be implicated as an important topic of study in the association between experiences of adversity and temporal discounting (as opposed to say structural disadvantage, e.g., lack of access to healthcare), and it does not yet clearly enough argue for why and how experiences of adversity in childhood should affect decision-making, above and beyond current experience.*

We thank the reviewer for these prompts for improvements. We believe this is clear and directive feedback and highlights a past limitation of the manuscript. In response to this comment, we have expanded the framing and introduction to better conceptualize individual decision-making as an important, though not sole, mechanism in the relations between socioeconomic disadvantage and later outcomes. Most notably, we highlight how socioeconomic disadvantage: 1) is associated with a myriad of other challenges and life stressors (lines 47-58); and 2) can influence behavior and brain development, influencing reward processing, executive functions, and their connected neural circuits (lines 81-95). We hope that this more richly motivated the work and improves the reviewer’s opinion of the manuscript. Of note, we did not provide an exhaustive review of these research areas as we did not actually have measurements of these constructs (e.g., stress exposure; fMRI responsivity/neurobiology); however, we are happy to further expand this framing if the reviewer thinks appropriate.

- *Despite framing the gap being addressed as a lack of understanding of the link between SEP and specific decision-making processes, the temporal discounting items used are undertheorized and underexplained – what is different in the gain, loss, and magnitude frames, and why are all three included? Why should we care about current income more so than other economic aspects of financial adversity (as Ruggeri et al., 2022’s paper on temporal discounting indicates we should)? What are the specific ‘choice anomalies’ tested for in the supplemental questions, and what can they tell us above and beyond the primary measures? How were the income titration percentages calculated, and why do they seem to use both participant income and national household average income? Why can we be confident in grouping together all of the measures into a single average (and is an average appropriate – i.e., would a factor analysis allow for a more appropriate weighting of the different components?) More theoretical contextualization is needed to explain why each temporal discounting measure should be included, what we should expect from each, and why they should be combined.*

We thank the reviewer for raising these issues. There are elements that we strongly agree with. There is also feedback that we believe provides an opportunity to clarify or strengthen motivation. Related to the primary measure of discounting that we used, we believe it is important to note that this is one of the first projects, to our knowledge, to examine delay discounting in adulthood and correlate it with childhood socioeconomic position. Given this fact, we believed that our results would most cleanly connect to the large body of delay/temporal discounting work that has looked at these measures in the aggregate (similar to our measures) and not with a more granular focus on choice anomalies. In addition, we have added clarifying language in the Methods section regarding the grouping of the different constructs and how the score was calculated (Pgs. 9-10). We hope these additional details will help to explain the robustness of the temporal discounting measure and address the reviewer’s concerns about the appropriateness of this measurement technique.

- *More attention could be paid in the initial framing of subjective vs. objective measures of childhood SEP. The measure of adult SEP is particularly problematic, as judgment of how things have changed cannot be used as a proxy for judgment of how one is doing overall (as it can make a poor person who did well in the pandemic look like they are higher in SES than a rich person who did poorly in the pandemic). The wording of this measure thus needs to be changed throughout, to something like ‘recent economic change’; alternatively, so that they can speak to something like adult SEP, the authors could use adult income in their analyses instead.*

We thank the reviewer for calling our attention to these areas for improvement and the lack of clarity on our description of childhood SEP measures. In connection to this comment as well as others raised by the reviewer about “objective” measures of childhood SEP, we have decided to move this set of analyses to the supplement as comparing objective and subjective measurements was not a main focus of the study. Regarding the wording of the adult SEP variable, we agree with the reviewer’s assessment and elected to change the variable name to recent economic change as suggested.

- *It seems that one of the key arguments is to push back on Ruggeri’s ‘positive deviance’ findings, but this is not mentioned in the setup. I would love a clearer articulation of the study’s theoretical framework, subsequent predictions, and if/how those predictions might prove important given the context of alternate findings in the literature.*

We thank the reviewer for noting this opportunity to strengthen the theoretical framework of the study and have done a complete restructuring of the introduction section to more clearly present the theory motivating our hypotheses. Some language remains the same, but we have included additional paragraphs (in lines 81-111) to further articulate the study’s theoretical framework.

- *Although preregistration of analysis of secondary data is only now becoming a norm, its absence here makes it harder to both 1) interpret how the analyses link to the theoretical framing, and 2) discount the role of researcher degrees of freedom. (E.g., a global temporal discounting score that combines the sub-variables would be more convincing if this analysis approach had been pre-registered. Without the pre-registration and especially without documentation of the related code readers are left wondering if the results hold only when the sub-items are combined).*

We appreciate this feedback and thought. However, given that analyses have already been completed, we are unsure how best to respond to this point. Our lab is doing more pre-registration for different studies, but the reviewer is correct that the project was not pre-registered. We have more clearly noted this on Pg. 6.

- *Relatedly, the lack of supplemental information means that the analyses reported cannot be checked, and greater detail on the measures and study sample used cannot be adequately considered.*

We apologize for this omission, we did not realize this was a necessary component for submissions to *PLOS-one*. In the revised submission, we have included additional details about measures and items, as well as data analysis.

- *Similarly, a greater discussion could be included as to why each method was selected, the reasoning behind how each method was conducted (e.g., more detail on the reasoning behind country-level random effects), and more reporting of missing values (why are missing values imputed?) and related robustness checks.*

We thank the reviewer for bringing this to our attention and agree that further reasoning for the use of statistical models is warranted. We have added clarifying language on Pg. 12 to explain why we chose linear mixed-effects models as well as the reasoning for including country as a random effect in the models. Furthermore, we have included additional information regarding our exploratory analyses included in the supplement. Regarding the use of imputation, we have moved this set of analyses to the supplement as referenced in an earlier comment.

- *As I understand it, the dataset being used was collected by other researchers, however this should be more clearly outlined (and the benefits and limitations discussed). It is also not clear from the original authors’ OSF file whether analysis of this data for use in future publications (as opposed to just for replication of the analyses in their paper) is permitted.*

We apologize for the lack of clarity here. We believe we noted this in our original submission, but the reviewer is likely correct that it is a detail that may be missed by readers. We have now revised the manuscript to more specifically emphasize this fact. In particular, a paragraph was added to the discussion (lines 377-387).

- *The use of existing datasets can be sufficient to warrant publication, however the current analyses underleverages the potential of the existing dataset. (E.g., getting beyond WEIRD samples is noted as a benefit in the Discussion section, but no analysis is made to consider if and how results vary according to country context such as country GDP and inequality).*

We appreciate this feedback and thought. In our supplemental models, we have now included a number of additional analyses including breaking effects down by individual country, comparing the results of our models in WEIRD versus non-WEIRD countries, and a strengthened explanation of the country-level analysis of global extreme poverty rate from the original manuscript. We believe these elements begin to unpack how country/context may influence our results. Of important note (in our supplement), we examined relations between discounting and childhood SEP in each country separately, running 61 statistical models with childhood SEP as the independent variable and discounting as the dependent variable. In these analyses, we find that participants in ~95% of the countries surveyed showed this association (see Supplement Pg. 2 and Figure S3).

- *If the paper is specifically pointing to limitations in existing research (Ruggeri’s ‘positive deviance’ findings), then it could more tightly point to limitations in that existing research and structure the analyses and discussion appropriately – as it stands I worry it is both too specific and too broad to meaningfully contribute above and beyond the original analysis.*

We appreciate the reviewer for raising this comment. In response to this critique, we had added discussion of Ruggeri’s ‘positive deviance’ findings (on Pg. 18). Specifically we noted that “*we analyzed the full range of childhood socioeconomic positions [..] Decision-making processes may continue to be negatively affected by childhood experiences well into adulthood, affecting responses to negative changes in one’s economic situation. This may be true even among those whose financial situation has improved since their early years”.* We believe this addition helps contextualize our findings in relation to past work by Ruggeri et al., but also a much larger body of research that looks at childhood SEP as a driving factor of disparities. This latter work does not typically focus on “deviances” between early and later social position, instead arguing that early social position directly and uniquely shapes later behavior, irrespective of later positioning and change.

- *While Figure 3 is compelling, greater discussion is needed to explain differences in the relationship between childhood SEP when measured continuously or discretely (Figure 4).*
- *As outlined in the discussion, the paper does not dig deeply into underpinning mechanism. As such it is hard to rule out alternative explanations, particularly given the cross-sectional nature of how the data are reported.*

We thank the reviewer for this comment. As noted above, we have expanded our discussion into how socioeconomic disadvantage: 1) is associated with a myriad of other challenges and life stressors; and 2) can influence behavior and brain development, influencing reward processing, executive functions, and their connected neural circuits. We believe this begins to move closer to mechanism; however, we want to be careful as to not go too far into this space (as we did not actually have measurements of these constructs). We have revised our introduction to help further motivate and contextualize the work (Pgs. 4-5). Regarding relations between childhood SEP when measured continuously or discretely, we have added a brief discussion in our manuscript’s supplement (Supplement Pg. 9). Specifically, we note that: “*we see that the lowest childhood SEP group shows greater temporal discounting than the multiple other groups (below average, above average). However, depending on the model covariates, there were variations in differences between the poor and other groups. This suggests that a continuous measure of social position may obscure non-linear relationships that become evident when analyzing this variable categorically. The variability in findings for the Around average v. Poor groups are surprising and do not want to overinterpret this null effect. For the highest (“Wealthy”) group, it is interesting to speculate that greater discounting may be seen in this group because of societal and structural privileges that may protect them from the negative consequences of greater discounting.”*

We thought it best to not overinterpret effects that were not hypothesized (connected to this reviewer’s other critiques about our lack of pre-registration), but wanted to be responsive to the issue raised here.

- *‘An interaction emerged between childhood and current socioeconomic status, such that the steepest temporal discounting was found for those facing persistent economic adversity across both time periods.’ – Are we talking about relative adversity or absolute adversity, if we’re comparing across countries?*

We thank the reviewer for raising this distinction between relative and absolute adversity. In response to this reviewer’s earlier feedback about the study’s theoretical motivation, we also reworded our hypotheses and interpretations to more cleanly connect to the more robust theoretical framework on stress sensitization theory. As a result, we no longer use this language to describe the results of our interaction model.

- *‘Findings provide new evidence that childhood poverty relates to more impulsive decision- making and steeper devaluation of future rewards later in adulthood, despite changes in economic mobility. This highlights poverty's persistent effects on core psychological processes underlying decision-making, suggesting early life socioeconomic position may have longer-term impacts on developmental trajectories.’ Careful with the language here – is it temporal discounting or impulsivity? Do we actually have purchase on psychological mechanism? How does it impact trajectories, and are we sure this is via decision-making?*
- *‘By elucidating developmental mechanisms linking childhood poverty to adult well-being, findings could inform policies aimed at narrowing socioeconomic gaps and disrupting cycles of disadvantage and economic marginalization.’ Interesting – I will be looking for more elaboration.*

We thank the reviewer for raising this comment. We believe they are indeed correct and we should temper our language due to the data that is available in this work. Said data does not speak to many of these issues of mechanism and trajectory, so we have tempered the language in the revised manuscript accordingly. Related to policy, we have included some additional speculation in our discussion (lines 365-377), primarily around societal and institutional trust. However, we are mindful of this reviewer’s broad comments about being “Careful with the language here”. Additionally, we have tried to revise the manuscript to avoid using synonyms or similar phrases for key variables of interest as to avoid confusion.

- *Line 51-52 – I want one more connecting phrase to explain why we should consider the role of decision-making in the connection between socioeconomic position and health*

We appreciate the reviewer’s attention to detail and thank them for offering this suggestion. As referenced earlier, we have done a thorough restructuring of the introduction. We hope the revised introduction offers a clearer picture of how decision-making connects to our discussion of socioeconomic position and health but remain open to further revisions related to this question.

- *Fairly broad in potential impacts – there is a tension here between wanting to pin down which types of decision-making are impacted, and drilling into a specific kind of decision-making (temporal discounting). I might pick one.*

We appreciate this note and have tried to reshape the manuscript to more crisply focus on temporal discounting by explaining the theory motivating our analyses as well as more specific justification for the examination of temporal discounting rather than a broad set of biases and choice anomalies (lines 203-209). In short, we favor temporal discounting as measured in the present study because it incorporates biases into the calculation (see lines 193-202).

- *Paragraph 2 (line 62) is interesting – I agree that childhood poverty is an important issue, but in some ways this undermines the argument about decision-making. Shouldn’t this issue be important enough that policy-makers take it seriously even if we don’t have clear links to decision-making? If it has such pervasive effects, why should we be interested in decision-making as opposed to other, more structural mechanisms through which poverty might affect outcomes (e.g., access).*

We thank the reviewer for raising this important consideration. It is not lost on us how important structural mechanisms are to shaping individual decision-making and agree that policymakers should care about childhood poverty regardless of whether or not there are links to decision-making. However, we disagree that this “*undermines the argument about decision-making*.” Although policymakers should care, not all of them do. Furthermore, less is known about residual effects of childhood poverty on cognitive and behavioral outcomes like decision-making. This connection between childhood socioeconomic position and economic choices in adulthood is not well understood, and we believe our results are important to informing policymakers about populations who may be especially vulnerable to economic stress such as the large-scale financial turndown experienced during the pandemic. To clarify this position, we have added greater detail about the theoretical motivation of our analyses in the introduction (see lines 81-111). We hope the revised introduction offers a clearer articulation of our argument.

- *Interesting that in paragraph one there are claims about us not understanding the link between SE position and specific decision-making processes, but then in paragraph 3 (line 73) widens the lens to temporal discounting as a catch-all (despite Ruggeri et al.’s 2022 article which breaks temporal discounting down into more specific components).*

We appreciate this note, but our team was slightly unclear of the specific critique here. We are presuming that this is similar to comments about “wanting to pin down which types of decision-making are impacted” versus “drilling into a specific kind of decision-making (temporal discounting)”. We appreciate this note and have tried to reshape the manuscript to focus more explicitly on temporal discounting. In the introduction, we have included more language explaining our focus on temporal discounting (Pg. 3), and in the revised methods section we also added further information about the temporal discounting instrument (starting on line 158) and how different biases are accounted for in the measurement technique (lines 185-192).

- *More work could be done in lining up why inequality should be thrown into the mix of potential mechanisms (89) – this comes on line 99, but it is strange to not have it before.*
- *Here we may need more reasoning as to why we should care about inequality at the level at which it is measured (vs. at a more local level), or more reasoning as to why national-level inequality is the closest proxy we can get in place of more local measures.*

We thank the reviewer for these insightful remarks and agree that our description of inequality in the context of this study is unclear. Generally, we are focused on the generalizability of the relationship between childhood socioeconomic position, recent economic change, and temporal discounting around the world. Thus, country-level inequalities are not a primary motivation of our hypotheses but are considered because of their plausible influence on our results. In the revised introduction section, we have attempted to reflect these priorities by listing country-level differences as a tertiary motivation. Furthermore, to ensure the data were utilized to their fullest extent, we have included an additional set of country-level analyses comparing WEIRD and non-WEIRD countries in the supplemental materials (see Supplement Pgs. 2-8).

- *It is unclear what is meant by ‘individual inequality’.*

We apologize for this confusion and agree that the terminology is unclear. Throughout the revision process, we reframed our analysis of inequality and included results strictly at the country level. They can be found in the supplement. We feel this may more accurately capture our aims and this will, hopefully, lessen confusion from readers.

- *Line 108 – greater explanation could be given as to what is implicated by stress sensitization, perhaps linking it in clearer language to the study at hand and relevant predictions. is? I would think that the difference would be between those who have experienced stress previously (and become sensitized to it) vs. those that hadn’t? (rather than those would have continued to be in a low SES position vs. Those that have experienced upward mobility)*

We thank the reviewer for noting this opportunity to strengthen the theoretical motivation for our analyses. In response to this comment and others, we have done a thorough restructuring of the introduction paragraph. We hope the revised introduction offers a clearer picture of the implications of stress sensitization theory and its connection to our predictions. However, we remain open to further revisions related to stress sensitization.

- *Final paragraph of introduction doesn’t go into detail on alternative mechanisms (e.g., the Jachimowicz et al. (2017)’s distinction between can you afford to wait, and do you trust that the thing you are waiting for will come to you?) – are we going to look at mechanism, or just verify the persistence of relationships?*

We thank the reviewer for noting this limitation in our writing. We have tried to add details to our introduction, that we are just verifying the persistence of relations. Given the lack of relevant data here, we obviously could not probe mechanisms; we however have added more details about this idea in our discussion section.

- *Participants – remind us of the representativeness of those individuals, particularly in non-WEIRD contexts? Are there limitations there in terms of who has access to Qualtrics (i.e., is this truly a random sample, or is this a broad sample that over-represents higher SES or higher educated individuals from non-WEIRD countries) – the education and student status stats make me wonder if the sample is truly representative, and the income stats (if it is cross-country) should be reported with a clearer unit (e.g., is income adjusted to USD?)*

We thank the reviewer for highlighting what we feel to be an important limitation of this sample that was overlooked in the submitted manuscript. As such we have added details (lines 388-395) in our discussion section about the sampling methods that may have contributed to a more educated sampling pool and acknowledges the sample may not be truly representative. In response to the reviewer’s questions about income, we have updated our Table 1 to reflect that income is, in fact, adjusted to USD.

- *Clarify that this does not include an objective measure of SES growing up and currently, but rather subjective measures, and that the current subjective measure is potentially distinct from more general measures of subjective economic position in that it specifically anchored to changes during the pandemic.*

We thank the reviewer for bringing our attention to this important clarification. In response to an earlier comment, we have more aptly renamed “current socioeconomic position” to “recent economic change” and added language in the procedure to emphasize both measures are subjective responses.

- *What percentage of the inequality data were imputed? (160) Are there robustness checks excluding the imputed data?*
- *For the inequality measure, I would like more explanation as to theorized mechanism in guiding which inequality measure was selected (childhood mortality and life expectancy I would expect to act quite differently from GDP or Gini wealth) – which measure here matters, and how is it theorized to impact decision-making?*
- *Similarly, I lost track of the reasoning behind the imputation and dataset pooling method – why not pick a single measure? What are ‘historical’ measures of ‘global inequality’ – are these measured in the same time period and at the same national level? If a single measure wasn’t picked, more detail is needed as to the reasoning behind the pooling methodology, and how it was conducted (or at least link us to the code).*

We apologize for the lack of clarity about the reasoning for using imputation to evaluate the global extreme poverty rate and subsequent expected results. As referenced earlier, we have decided to move this segment of our analysis to the supplement. This was due to potential confusion (of objective versus subjective childhood SEP), the lack of significant results (in a well-powered sample), and these issues with data missingness. To answer your question, approximately 2.7% of the data were imputed due to systematic missingness among participants from certain countries. We have detailed this set of analyses in the supplement (Supplement Pgs. 5-6). and made all of our code available on our OSF site.

- *Why the gain, loss, and magnitude frames? This needs more theoretical contextualization to explain why we should include each, and what we should expect from each.*

We thank the reviewer for identifying this area to more clearly describe the motivation for including multiple frames in our calculation of temporal discounting. In the revised manuscript, we have restructured and rewritten the “Temporal discounting” section of our procedure, adding language detailing our theoretical motivation in the introduction and how questions differed between frames. However, we are reluctant to hypothesize about the expected results from each frame as we did not analyze these data and are primarily interested in individuals’ tendencies to choose smaller, sooner rewards above and beyond how such decisions are framed.

- *Why calibrate to the participant’s annual income? Here it would be helpful to either explain the reasoning or to cite someone else who has previously used this methodology.*

We thank the reviewer for catching this error in our description of the temporal discounting instrument. Binary choice values in the temporal discounting task were calibrated to “approximately 10% of the national household income average (either median or mean, depending on the local standard) of the participant’s country of residence.” We apologize for the error and have revised the section to more clearly and accurately articulate how choice values were calculated.

- *What is a TD score? (175) – Total discounting? It might be worth spelling it out the first time it is referenced.*

We apologize for this omission and have corrected this in the revised manuscript on lines 193-195. Thank you for catching this error.

- *Similarly, what are the supplemental questions? (175) What do they tell us that we do not already have in the previous scores, what do we predict they will show, and how should they be interpreted? What specifically is meant by a ‘choice anomalies’, and why does analysis of specific anomalies fall beyond the scope of this work? (180) Was this pre-registered?*

We apologize for this omission and have included a paragraph in the revised manuscript describing the supplemental questions in detail (lines 185-192). However, as with the gain, loss, and magnitude frames we are reluctant to hypothesize about results that we did not analyze. We hope the first paragraph of the revised temporal discounting procedure as well as our restructured introduction offers a clearer picture about why frames and different choice anomalies are not of interest here.

- *How do we access the supplement? (184) Why then on 412 are there ‘no supplementary materials to provide’?*

We apologize for the omission and lack of attention to detail in citing supplementary materials that were not provided. In the revised manuscript we have included a robust set of additional analyses and information regarding the temporal discounting score as was referenced here.

- *How was a gain of 10% decided upon? Why a time period of a year? (189)*
- *Similarly, why 120 then 150? (vs. 102 and 101?) (193)*
- *Did I understand this correctly, that the discounting choice value was based on participant reported annual income (as specified in line 171), and then the increase and decrease in subsequent questions was based on the national household average income? (187) If so, why? (E.g., are personal and household incomes comparable? Are choices still equivalent at different points along the income scale, where those with lower relative starting incomes will be asked about a greater relative gain vs. those with higher relative starting incomes – i.e., those of lower socioeconomic position are receiving a different question insofar as they are being ask to choose between options with relatively greater gains and losses, as compared to their higher income peers?)*

We thank the reviewer for this question and apologize for the lack of clarity in our explanation of the temporal discounting instrument. The reviewer is correct that our original manuscript cited participant annual income as the initial discounting choice value. However, this was an error on our part. We have revised the temporal discounting procedure section to more clearly and accurately articulate how choice values were calculated (lines 193-202). We hope our revisions are adequate in addressing the reviewers questions. In terms of specific choices about discounting scenarios and subsequent choice sets, those were made by Ruggeri and colleagues. However, many past cross-national projects suggest economic changes as low as 10% can be perceived (and “felt”) by an individual. Other research teams suggest this threshold is closer to 25% (depending on the baseline economic conditions and the country sampled). These include work by Bonke & Browning, Easterlin, and Hagerty, & Veenhoven. Ruggeri and colleagues likely went to this lower threshold (~10%) to have greater sensitivity to changing reward valuations. We are happy to add these details to the main manuscript or the supplement if the reviewer thinks appropriate.

Example Work (Cited):

-Bonke, J., & Browning, M. (2009). The distribution of financial well-being and income within the household. Review of Economics of the Household, 7, 31-42.

-Easterlin, R. A. (1995). Will raising the incomes of all increase the happiness of all?. Journal of economic behavior & organization, 27(1), 35-47.

-Hagerty, M. R., & Veenhoven, R. (2003). Wealth and happiness revisited–growing national income does go with greater happiness. Social indicators research, 64, 1-27.

- *Overall – I would recommend bulking up the reasoning behind each analysis. (What question is it meant to answer, and why is the type of analysis selected best? Are there alternative explanations or robustness checks that can be tested?)*
- *Why use an LMEM model? (Do we expect effects to be linear?)*

We appreciate the reviewer’s attention to detail in evaluating our statistical analyses. As noted earlier in response to a previous comment, we have rewritten the introduction to ground the paper on a stronger theoretical foundation with clearer connections to our analyses. Furthermore, we have included additional clarification regarding the use of LMEMs in the methods section (see Pgs. 11-12). We hope that the revised manuscript better illustrates the connections between theory and our analyses.

- *Make more explicit why country of residence has a random effect but not the other variables (e.g., age).*

We thank the reviewer for citing this opportunity to more clearly explain the choice of random effects in our models. Country of residence was chosen as a random effect based on general intuition and initial exploratory analyses which suggest that the relationship between childhood socioeconomic position and temporal discounting varies by country. We have included language in the revised statistical modeling procedure that explains these analyses and cites supplementary figures to support this intuition (see Pgs. 11-12)

- *Are these planned analyses (215) pre-registered?*

The project was not pre-registered. We have noted this on Pg. 6.

- *Why are there multiple analyses, and how do they relate to one another? (What will each of the different analyses give us insight into and are they testing variations on the same hypothesis – i.e., how should we think about correction for multiple hypothesis testing?)*

We thank the reviewer for this important question. As noted earlier, we have revised the paper to more cleanly connect our theoretical motivation to our hypotheses and, in turn, to our analyses. We hope the revised introduction illustrates that we are testing two main hypotheses that while related are distinct from one another. Developmental origins of disease theory motivated the first set of models which test for a main effect of childhood socioeconomic position on temporal discounting in adulthood. On the other hand, stress sensitization theory motivated an interaction model that tested for

- *What do we make of the correlations being so low?*

This is an interesting point, but we feel a bit more nuanced than the reviewer is implying. Specifically, we think it is important to: 1) Think about what a meaningful effect (size) is; and 2) Publish research (that is well-executed) regardless of the effect size.

Regarding the first point, how meaningful is an effect size is an area of active debate and exploration across the social sciences. As thoughtfully articulated by Bakker et al. (2019): *“One important aspect of situating the research is realizing that the role and interpretation of effect size will depend on the goal of the study”.* The goal of our work was to see if there were relations between child socioeconomic position and later behavior. We find support for this idea that has not been previously examined. Notably, we leveraged a large, multi-national cohort and found these associations across many countries. While the child socioeconomic position was retrospective, it was a proxy for experiences that happened decades earlier for participants. Finding support for that idea is notable in our opinion as there are many intervening factors (from childhood to adulthood) and there are likely heterogeneous subgroups within our sample. It would be very interesting to explore if childhood socioeconomic position interacted with other (intervening) factors to predict later adult behavior. We think this is a great direction to pursue in future projects. However, noting long-term influences on behavior is an important contribution to the literature (and a goal of our work). Related to the second point, we have thoroughly detailed the magnitude of our effects (in terms of t-statistics, p-values, and effect sizes), we think this will allow *PLOS-one* readers to judge for themselves about the influence and impact of the relations that we reported.

Work Cited:

-Bakker, A., Cai, J., English, L., Kaiser, G., Mesa, V., & Van Dooren, W. (2019). Beyond small, medium, or large: Points of consideration when interpreting effect sizes. Educational Studies in Mathematics, 102, 1-8.

- *Table 2: It would be helpful to have a breakdown of the study demographics according to these categories. (e.g., what percentage of the sample listed Gender as Other? What percentage was retired? Etc.)*

We thank the reviewer for bringing attention to these important characteristics and apologize if our presentation of the sample demographics was unclear. The full breakdown of the sample by category including percentages is given in Table 1. If the reviewer has specific recommendations for a more effective method of presenting these statistics we are certainly open to accommodating them.

- *Why do a hierarchical regression but only for education completed (and not the other demographic variables like gender and employment?) If we’re interested in Childhood SEP I might run the model looking just at that and then see if it holds when controlling for potential alternative explanations. (But tell us why each of the control variables is important to include).*

We apologize for the lack of clarity and thank the reviewer for this question. It seems as though the reviewer misunderstood our initial set of analyses. We ran two hierarchical regressions to probe a main effect of childhood socioeconomic position on temporal discounting. Both included demographic variables such as age, gender, and employment status, but one model includes education completed and the other does not. This was done because the sample is highly educated, and we have noted this reasoning in the revised manuscript (lines 389-396). As for the other control variables, we have also added language in the statistical modeling procedure explaining the use of each covariate in our model (see Pgs. 11-12).

- *What should we make of the extremely small estimates for childhood SEP? How can this be interpreted in real terms? (Even if the association is significant, is it meaningful?)*

As noted above, while child socioeconomic position was retrospectively reported, it was a proxy for experiences that happened decades earlier for participants. Finding support for that idea is notable in our opinion as there are many intervening factors (from childhood to adulthood) and there are likely heterogeneous subgroups within our sample. It would be very interesting to explore if childhood socioeconomic position interacted with other (intervening) factors to predict later adult behavior. We think this is a great direction to pursue in future projects. However, noting long-term influences on behavior is an important contribution to the literature.

- *For the interaction probe, how is current SEP calculated? (266)*

Based on the reviewer’s suggestion in an earlier comment, we have renamed the “Current SEP” variable to “Recent Economic Change.” We agree that this is a more appropriate title for the variable which is categorical and represents each participant’s response to the question, “Overall, and considering the global pandemic, how did your financial situation change during 2020?” We have included a separate section in the methods section to help clarify this calculation (see line 148).

- *Figure 3 – if we’re interested in the effects of childhood SEP, I wonder if it wouldn’t be more intuitive to look at current SEP with lines breaking down childhood SEP SDs.*

We thank the reviewer for this suggestion and have changed our figures accordingly to break down recent economic change by standard deviations rather than childhood SEP.

- *Explain why is it important to look at childhood SEP categorically. (282) (E.g., Because it is measured on a Likert?)*

Yes, the reviewer is correct. This dataset only permits analysis of childhood SEP measured based on self-report which was coded to a 5-point Likert scale. We apologize for the lack of clarity about this measurement and have updated the methods section to include a sentence explicitly stating the use of a Likert scale and the correspondence of each response on the 5-point scale.

- *Why is the absence of any main effect of country-level inequality justification for not looking for an interactive role for inequality?*

We thank the reviewer for raising this important consideration and agree that this important strength of the dataset (i.e. multinational) was under-analyzed in our initial analysis. As noted earlier, the country-level analysis of inequality (global extreme poverty rates) was moved to the supplement and removed from our earlier models. Thus, we have reframed our interpretations of these results and no longer make the claim that the absence of a main effect precludes the investigation of an interaction.

- *The sample is multinational – I was curious to see more breakdown by country GDP, country inequality (especially given the intro about inequality?) etc.*
- *This breakdown would be particularly useful to inform discussion of WEIRD differences in the Discussion section (311).*

We appreciate the reviewer raising these important points about country-level factors. Our revised introduction includes tempered language about inequality, as it is less of a focus than was reflected in our original manuscript. However, we agree that not enough was done to leverage this multinational cohort. In response to these and earlier comments, we have included a forest plot breaking down main effects by country as well as a new set of analyses looking at WEIRD and non-WEIRD analyses.

- *Description of the results of the simple slopes analysis is inadequate: ideally this should break down the interaction to compare either the effect of adult SEP at different levels of childhood SEP or vice versa, not just say which group did the most discounting.*

We thank the reviewer for catching this oversight. On lines 293-300, we have included a full interpretation of the interaction effect between childhood SEP and recent economic change on temporal discounting.

- *Careful with the wording (303) – this does not show us childhood socioeconomic position, just current perceptions of childhood position.*

We thank the reviewer for highlighting this point of potential confusion for readers.

- *Where does reported financial hardship come in if we have reported annual income? How should we understand differences between subjective report (which might be more susceptible to something like method variance in pessimism) vs. more ‘objective’ measures like reported income? For ‘objective’ measures, what are their strengths and limitations? (e.g., is income the best variable to measure economic situation, particularly given Ruggeri et al.’s (2022) findings about different aspects of financial hardship being important predictors of temporal discounting?)*

We appreciate the reviewer raising these important methodological concerns but feel as though they fall well beyond the scope of the present study given we did not analyze reported annual income. If the reviewer has further suggestions or comments about how these ideas connect to the analyses we did conduct, then we are more than happy to look into them.

- *Could this work be similarly linked to population choice patterns, but considering childhood rather than current financial status? (313)*
- *331 In the discrete version a relationship was not found with discounting- how can this be explained?*
- *338 What predictions? These should be more clearly outlined in the setup.*

We thank the reviewer for raising these comments. Regarding linking to population choice patterns, we think this is an interesting idea; however, it feels outside the scope of our work. We are trying to balance the elements that the reviewer is seeking here with other critiques from this scholar where we are reminded to temper our language and that our relations are small. Given the lack of findings with population-level variables (in the supplement), we think it too early to link our work to population choice patterns (and consider childhood rather than current financial status). However, if the reviewer thinks appropriate, we are happy to provide more discussion and speculation in the manuscript. We would only ask for a bit more clarity as this comment was a bit challenging to parse.

Related to discrete versus continuous relations, we don’t see these effects as being inconsistent. It is perhaps more about potential “threshold” effects and childhood SEP exerting influences at certain levels. We discuss this in our supplement (Supplement Pg. 9).

- *Careful with what work is cited – The example on 349 doesn’t illustrate the point in 347 (i.e., perceived unsafety vs. perceived safety does not give us clear purchase on the relative robustness of subjective vs. objective measures).*

We thank the reviewer for bringing our attention to this aspect of our works cited. With the country-level analysis of global extreme poverty rate (previously referred to as the “objective measure” in our original manuscript) moved to the supplement, this discussion has been omitted from the revised manuscript.

- *The pushback on Ruggeri’s ‘positive deviance’ findings is interesting, but it does not address his work that finds biases or less ‘rational’ temporal discounting choice is not the purview only of those experiencing financial hardship (see Ruggeri et al., 2022).*

Thanks to this reviewer for raising this comment, we have included further discussion of this work and different biases in our manuscript related to this critique (Pg. 18).

We appreciate your consideration of this manuscript and hope that with the improvements suggested by the reviewers, the manuscript is acceptable for publication.

Sincerely,

Jamie L. Hanson

Assistant Professor

Department of Psychology

University of Pittsburgh
